# Evaluation of Synchronous and Asynchronous Telemedical Applications in Primary Care in Rural Regions of Northern Germany—Results and Lessons Learned from a Pilot Study

**DOI:** 10.3390/ijerph192214860

**Published:** 2022-11-11

**Authors:** Alexander Waschkau, Pia Traulsen, Jost Steinhäuser

**Affiliations:** Institute of Family Medicine, University Medical Center Schleswig-Holstein, Campus Lübeck, Ratzeburger Allee 160, 23562 Lübeck, Germany

**Keywords:** primary care, family medicine, telemedicine, eHealth, rural, asynchronous telemedicine

## Abstract

(1) Background: Telemedical applications (TAs) that are centered around General practitioners’ (GP) practices could be beneficial for patients in rural areas in order to better their access to care. This could become more and more relevant as specialists favor practicing in more urban regions, leaving GPs as the first medical contact of patients in rural areas. (2) Methods: Three TAs, one synchronous, one asynchronous and one used in delegation were implemented and evaluated in ten GP practices and two specialists’ practices in rural areas of northern Germany. (3) Results: Overall satisfaction with the TAs was generally high. GPs as well as specialists were especially satisfied with asynchronous TAs. A number of valuable “Lesson learned” were obtained and can be used as recommendations for further studies, e.g., taking time to identify market-ready technologies prior to implementation, developing dedicated trainings for users, and preparation of a technical support plan. Overall, the benefits of the TAs were rated high for the patients by the medical professionals. (4) Conclusion: Especially asynchronous TAs that are based on existing technology can be successfully implemented into a developing digital health care system such as the one in Germany. The impact on treatment of those TAs needs to be further investigated.

## 1. Introduction

Reports of local shortages of general practitioners (GPs), which will make it difficult to ensure close-to-home care of patients, especially in rural areas, have internationally been an ongoing topic in recent years. Such a shortage is also being identified in some rural areas in Germany. GPs represent an essential factor in a community [1]. They are often the first medical contact for patients in the context of medical care. It is known that GPs can resolve up to 90% of all consultations in their practice without relying on further expertise of other medical professions. For the remaining 10% of consultations, they function as coordinators—a crucially important role [2]. For some time now, a primary care system has been deemed beneficial for the increasing number of patients with complex illnesses. Patients benefit from specialists e.g. dermatologists or rheumatologists staying in contact with the GP to avoid breaks in the information chain [2]. However, specialists in Germany tend to settle in urban areas above 20,000 inhabitants [3]. This leaves patients in rural areas with the dilemma of covering longer distances in order to reach those specialized practices.

In this situation, telemedical applications (TA) could be an additional care option to improve access to care. A 2018 survey showed that using telemedicine was conceivable for nearly 50% of patients, especially when they were guided by a nurse or doctor while using the technology [4].

Although there is already a large body of results on TAs among GPs on the international level [5,6], Germany is still in the early stages of implementation. A recent comparison of the implementation of eHealth applications in the European Union (EU) has shown that Germany fell from ranking 14th in 2013 to 17th in 2018 of 28 countries [7]. Around three quarters of all doctors in Germany see digitization as an opportunity for the health system, the excess of regulation in Germany is seen as one of the reasons for the slow progress [8]. Another reason could be the attitude of the National Association of Statutory Health Insurance Physicians (KBV), which, in its evaluation of a survey of doctors in 2021, comes to the following conclusion: “Only digital applications that have been comprehensively tested in the field may be adopted across the board in the provision of care” [9]. Since the KBV is responsible for the remuneration of these services, corresponding studies are necessary to pave the way for TAs to become part of standard care in Germany.

Therefore, TAs that were centered around the GP’s practice were developed and implemented in this pilot study. We decided to evaluate one synchronous, one asynchronous and one application that is used during delegation. To ensure that not too much time was spent on developing the applications, it was decided to use existing technologies. In a first step, these technologies were subjected to a feasibility analysis in which barriers and facilitating factors for the applications were identified [10].

### 1.1. Synchronous Video Liaison Consultations of GPs with Patient and Ophthalmologist

Cooperation models for the collaboration of physicians of different specialties together with a patient were recommended under the term “liaison consultations” as a solution to arising challenges in primary care in 2009 [11]. Since then, this approach has not been implemented across the board. The COVID-19 pandemic has boosted the video consultations between physicians and patients, but video liaisons between physicians of different specialties are still very rare [12]. Since the aforementioned preliminary study showed the willingness of the citizens to contact an area specialist is highest together with the family doctor via telemedicine, it could be theorized that such telemedical liaison consultations could be implemented nationwide [4]. In rural areas, this kind of telemedical liaison consultation could ideally spare patients both longer travel times to the local doctor and waiting times for an appointment with a specialist. One could also assume positive effects for the doctors involved. The specialist is, under certain circumstances, enabled to solve the consultation quickly and with the help of queries to the GP without having to physically see the patient in his or her practice. For the GP, there is the possibility to gain more security in situations where the diagnosis is unclear, thus would be able to expand their own knowledge. Overall, video liaison consultations could be an opportunity to improve patient care in a resource-efficient manner.

To smoothly implement video liaison consultations in general, we choose several stages of preliminary tests [5]. In the first phase, rare reasons for consultation-in our case ophthalmological questions-are to be carried out using this TA. This was done to slowly develop a routine with this new form of communication between GP and specialists in the presence of the patient without interrupting the daily organization of the practice too much. The idea was to gain important experience as to how the video liaison consultation can be integrated into everyday practice before a more frequent use could be undertaken.

### 1.2. Asynchronous Teledermatological Consultations

Dermatological concerns are, with 15%, one of the most common reasons for consultation in general practices in Germany [13]. Teledermatological applications have existed for over 25 years [14]. A distinction is made between three forms of teledermatological consultation. Synchronous video transmission, asynchronous transmission of images as well as hybrid forms in which images are transmitted and the dermatologist is able to ask further anamnestic questions via video conference [15]. International studies suggest that the diagnostic accuracy of teledermatology is high when compared to face-to-face diagnostics [16,17]. In terms of cost reduction and reduction in referrals, a study examining over 37,000 individual teleconsultations showed savings of 18%. The GPs involved reported that in 85% of the cases the consultation with the dermatologist had a positive effect on their knowledge and qualification in dealing with dermatology consultations [18]. At the time of the project onset (July 2019), dermatological care using telemedicine was not commonly implemented nationwide in Germany. 

### 1.3. Usage of Digital Vital Sensors by a Medical Assistant during Home Visits

In Germany, medical assistants of GP practices (Medizinische Fachangestellte, MFA) are allowed to perform delegated home visits. In order to do so, they have to acquire a specific additional qualification. GPs generally are willing to delegate standardized tasks to an MFA [19]. Delegated home visits help doctors reduce their working hours and increase the number of their patients [20]. Usually, the MFA documents the measured vital data of patients during their home visits and transfer those data at the end of the home visit tour to the patient record in the GP’s practice by hand. The GPs are thus usually able to assess the patients’ vital data only when the MFA is back in the practice. It seemed interesting to evaluate whether a more direct data transfer with the help of telemedicine could be helpful for the GPs and thus, of course, for the patients also.

TAs were chosen on the basis of availability of technology and their expected frequency of usage from rarely needed (ophthalmological), to sometimes needed (dermatological), to regularly used (delegated home visited). Another factor was the intrusiveness into practice processes, which was expected to be negatively correlated with the expected frequency of usage. Ophthalmological liaisons were rated as highly intrusive, because patients had to stay in the practice until a connection with the ophthalmologist was established, dermatological liaisons were rated mildly intrusive, and due to the fact that the MFAs operated outside of the practice this TA was rated minimal intrusive.

The aim of this pilot study was to implement synchronous and asynchronous TAs into daily GP care and evaluate facilitators and barriers in the implementation, as well as documenting in which cases these applications were used. The main focus of this pilot study was therefore to assess feasibility, needed organizational changes and satisfaction of users. Since doctors and MFAs in Germany still have little experience with this form of care, we decided to initially survey only the satisfaction of this group. A direct comparison of the three applications was not intended.

## 2. Materials and Methods

### 2.1. Synchronous Video Liaison Consultations of GPs with Patient and Ophthalmologist

Research practices were equipped with the necessary hardware (video telephones, webcams and loudspeakers) and GPs had the opportunity to request a video liaison consultation from an ophthalmologist if the need for a specialized liaison during the treatment of a patient was identified. Process evaluation of these video liaison consultations included the manageability and functionality of the technical devices in order to identify determinants for use in standard care.

### 2.2. Asynchronous Teledermatological Consultations

The research practices received tablets equipped with special lenses including a clip-on light. Practices could also use smartphones as well, since the quality of smartphone cameras was identified as perfectly adequate in 2019. Images of the skin should be taken in ambiguous cases in the GP’s practice. Those pictures were then sent to a dermatologist accompanied by a specialized documentation sheet via a data protection-compliant messaging app, after patients gave their written consent. The dermatologist agreed to provide feedback concerning the cases within 48 h.

### 2.3. Usage of Digital Vital Sensors by a Medical Assistant during Home Visits

For the pilot study, MFAs were specially trained to carry out digital measurements of vital signs during a home visit. For this purpose, they were equipped with a set of digital vital sensors. This included a tablet, a pulse oximeter, a scale, a blood pressure monitor, a spirometer, a blood glucose meter, and a 3-lead ECG. If the MFA needed medical support, they had the option of contacting the GP via a video conference using the tablet. Patient data were recorded and then transferred from the tablet to the practice in an encrypted form via mobile internet. The data could then be imported directly into the patient records in the GP practice. Each usage of the digital vital sensors should be documented for the ongoing process evaluation.

### 2.4. Duration of the Study and Used Equipment

From July 2019 to June 2021, ten GP practices, one ophthalmology center and one dermatology practice were equipped with technology for the TAs. Each practice received a videotelephone. All of the ten GP practices were located in rural areas (some were even located on islands in either the North Sea or the Baltic Sea) in the northernmost federal state of Germany, Schleswig-Holstein. Schleswig-Holstein has three million inhabitants. The practices were recruited from the pool of scientific collaboration practices of the Institute of Family Medicine, Lübeck, as well as via two political active GP associations that were partners in the project. Of the ten GP practices, nine were equipped with the vital sensors for usage during home visits by the MFAs (one practice, located at a small island had no need to use it). All personnel were trained in the usage of the TAs which included the possibility of an asynchronous teledermatological consultation. At the beginning of the study, every practice as well as the specialists received extensive training in the application. Training materials were prepared in advance of the study and a research assistant was available to provide technical support for the duration of the study. 

### 2.5. Evaluation

A custom developed paper-based documentation form was used for the video liaison consultations and usage of vital sensors during a home visit. Users (GPs/MFAs) were instructed to complete these forms after each consultation/use. Completion time was estimated to be between three to five minutes.

#### 2.5.1. Shared Items in the Evaluation of TAs

For both applications (video liaison consultation and vital sensors), the following data were collected:

- gender of patient;
- year of birth of patient;
- reason for the consultation;
- result of consultation;
- doctor’s/MFA’s assessment of the benefit for the patients.

Both documentation forms offered the possibility to add free-text comments.

#### 2.5.2. Unique Items in the Evaluation of the Synchronous Video Liaison Consultations

The quality of treatment was rated on a five-point scale with the answer options “very high”, “high”, “part-part”, “low”, and “very low”.

The quality of contact via video liaison consultations was rated in comparison to a face-to-face contact with the three answer options “worse”, “as well as”, and “better”.

Organizational execution, operation of the videotelephone, transmission quality and the consultation overall were rated on a three-point scale with the options “satisfactory”, “part-part”, and “unsatisfactory”.

#### 2.5.3. Unique Items in the Evaluation of the Digital Vital Sensors by an MFA during Home Visits

The time in hours after which the GP visited a patient was documented, if such a visit was deemed necessary. If the GP had to visit a patient, the added value of the visit was rated on a five-point scale with the options “very high”, “high,” part-part”, “low”, and “very low”. The certainty with which the home visit could be successfully completed by the MFA without a subsequent visit by the GP should be rated from zero to 100%. For each of the six digital vital sensors the usage was documented. If there was a need for a video conference with the GP, the reason for the video conference and the duration could be documented. The overall satisfaction with the set of digital vital sensors and the video conference was rated on a three-point scale with the options “satisfactory”, “part–part”, and “unsatisfactory”.

#### 2.5.4. Documentation of Asynchronous Teledermatological Consultations

Evaluation of the teledermatological cases was performed using the documentation forms that were used by the GPs and dermatologist.

## 3. Results

### 3.1. Video Liaison Consultations of GPs with Patient and Ophthalmologist

Overall, 17 video liaison consultations were documented during the course of the study. For further details see Table 1. The average waiting time between the identification of the need for a video liaison consultation and the actual start was eleven minutes, with the shortest waiting time being one minute and the longest 45 minutes. The explanation for the waiting time is as follows: After receiving the info that a video liaison consultation is necessary, the first available ophthalmologist, after finishing their current patient, started this liaison consultation via video telephone. If the ophthalmology center was very crowded or had emergencies, the waiting time for the GP and their patient was extended. Video liaison consultations themselves lasted between three and ten minutes with an average duration of six minutes.

Among the reasons for video liaison consultations were reddening of the eye, styes, and supposed allergic reactions. One of the more uncommon reasons was the loss of eyelashes in one case. GPs documented that video liaison consultations were helpful for patients in 94% of the cases. Referral to the specialist’s practice was necessary in three of the seventeen cases. The ophthalmologist involved confirmed that the reasons for the consultations were typical for referrals from GPs and could be treated very well by video liaison consultation, as they primarily concerned impairments of the outer eye.

In 71% of the cases, the consultations were rated “as well as” or “better” than the direct personal contact by the doctors. The operation of the video equipment was rated as satisfactory by 82% of doctors. The quality of transmission was rated as “satisfactory” by 94% of doctors. The overall satisfaction with the video liaison consultation was rated as “satisfactory” by 94%.

### 3.2. Asynchronous Teledermatological Consultations

During the time of this study, 24 teledermatological consultations were carried out. 

The duration between the request for a consultation and recommendation by the dermatologist was between 10 h and 145 h with an average of 47 h. Suspected diagnoses were:

- Local reaction after animal bite ×2.
- Atopic eczema ×2.
- Mycosis ×2.
- Stasis dermatitis.
- Scabies.
- Herpes-like infection.
- Skin lesion nose.
- Dermal scar.
- Urticaria.
- Rosacea or acne.
- Varicella zoster.
- Majorca callus.
- Alopecia areata.
- Itching, knee + left thigh.
- Eczema left wrist.
- Redness, swelling right wrist.
- Unclear ×5.

Based on the available documentation the suspected diagnoses were confirmed in nine cases (38%). The dermatologist involved confirmed that the cases presented during the asynchronous telemedicine consultations were unusual and indeed warranted further expertise from a dermatologist. During regular contacts with the study management, both the GPs and the dermatologist stated that they were satisfied with this type of care, after an initial inquiry by the dermatologist to better the quality of the accompanying photos. The improvement of photo quality involved a better lighting situation and usage of other angles that helped in assessing the size of skin lesions. No further technology was needed. The unclear cases were either identified by the dermatologist or resulted in the recommendation of referral to a dermatologist. 

### 3.3. Usage of Digital Vital Sensors by an MFA during Home Visits

The usage of vital sensors by an MFA during a delegated home visit was documented 158 times.

Reasons for the delegated home visit were primarily the measuring of blood pressure in 112 cases followed by “routine visits” in 36 cases. In seven cases, the reason for the visit was the taking of a blood sample and in three cases it was both measurement of blood sugar and doing an ECG. Individual mentions included the visit after a stationary treatment in hospital or bed sore documentation.

In four cases, a visit by the doctor was necessary after one, three, six and 24 h after the delegated home visit by an MFA. The reasons for these visits by the doctors were in two cases the results of blood pressure measurement. In one case, an ECG had to be written because of arrhythmia and in one case there were symptoms of acute chest pain and high blood pressure, which finally led to the patient´s hospitalization. Overall, the certainty with which this home visit could be successfully completed by the MFA even without a subsequent visit by the doctor was rated very high by 93% of the medical assistants.

**Figure 1:**
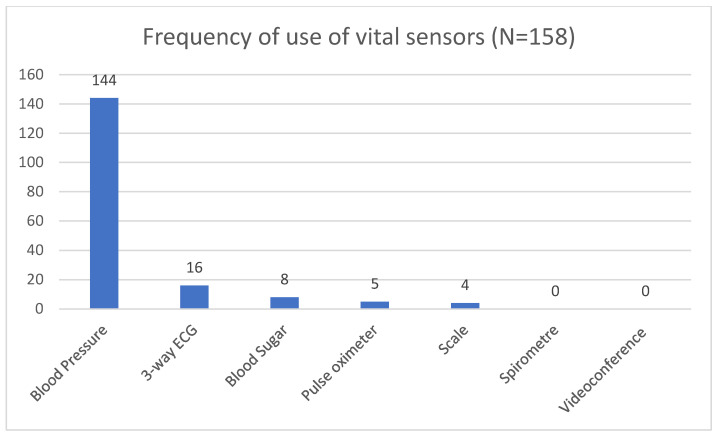
Frequency of use of the vital sensors.

The usage of the available vital sensors varied, as seen in Figure 1. Due to the possibility of more than one sensor being used during a home visit the total number of usages is higher than the number of visits.

This TA was “satisfactory” for 83% of MFA. Reasons to not be fully satisfied were primarily the lack of mobile internet in the rural region of the home visit or technical difficulties of the system as such.

MFAs documented that patients profited from these home visits with digital vital sensors in 86% of the cases.

## 4. Discussion

The aim of this pilot study was to implement and evaluate three TAs, that focused on intersectoral connections of GP’s practices with specialists. 

Specifically chosen were those applications which did not require the development of new technology. Concerning the software, only existing solutions that were legally approved for the health market in Germany were used. This was done in order to be able to quickly give recommendations for possible widespread use in case of a positive evaluation. A fair amount of time was therefore set aside prior to the study in order to identify those technologies and develop organizational changes for the practices to implement the TAs [10]. To implement new technologies and thus also change organizational steps in existing structures, it is inevitable to follow a standardized pathway. Otherwise, studies of the past have shown significantly less success in implementing new technologies in care [21].

Still one of the most relevant aspects of TAs is the availability of broadband internet [22]. The lack of broadband internet in some rural regions of the northern federal state Schleswig-Holstein, Germany, was one of main reasons for not using all three applications. There was one practice, e.g., that became a heavy user of the teledermatological consultation because it was possible to use it via a smartphone but was unable to use the video liaison consultations due to the insufficient internet performance in the practice, which was due to the lack of availability of a faster connection.

Poor mobile network coverage also proved to be a barrier for the usage of the digital vital sensors that were used by an MFA during their delegated home visits. Germany is ranked 57th in the availability of 4G in a global comparison and this becomes evident especially in rural areas [23]. If the direct transmission of patient data into the GP’s practice is blocked by non-existing mobile internet, the benefits of this kind of care are lost. This finding might be one of the reasons why the company that developed this system has stopped this service in the meantime.

In international literature, home telemonitoring is usually operationalized by measurements taken by patients alone with equipment provided. The effectiveness is often inconclusive, and costs are usually high [6,24,25,26]. We specifically tried to implement another way of home telemonitoring, which seemed very feasible in obtaining data and was derived from prior studies [4]. If this method has any effects on the treatment of patients, it has to be addressed by further studies.

Due to the fact that this pilot study took place during the COVID-19 pandemic there was a reported decline in the number of in person visits by MFAs. The main concern was protection against infection, as not enough protective clothing was available at all times [27]. It could be argued that the decline of more than 12% of consultations in the GP practices could have had an influence on the number of usages of the TA [28]. The uptake of video consultations with patients in general had little to no influence on the usage of the synchronous video liaison consultations with ophthalmologists [29]. This might be due to the fact that patients chose more specific reasons for visiting their GP and tried directly to visit an ophthalmologist when they had concerns about their eyes.

Another reason why some TAs were not used was the individual regional situation. If there was an ophthalmologist or dermatologist in the immediate vicinity of the GP practice, the corresponding TA was not used. This factor is relevant in order to understand all the variables that could impact the implementation of TAs. 

While teledermatology is a common practice in many countries around the world, the lack of reimbursement for this kind of care in Germany explains the novelty for practices and the slow uptake [30]. It was a challenge to identify a dermatologist that was willing to invest time and expertise, with only one case in the study being reimbursed through a special contract with one health insurance company in Germany. The Association of Statutory Health Insurance Physicians in the federal state of Baden-Württemberg in Germany (KVBW) initiated a two-year exclusively remote care service for statutorily insured persons (docdirekt). Baden-Württemberg has ten million inhabitants. In the two years, 3090 cases were treated telemedically [31]. It seems like German patients prefer face-to-face consultations whenever possible.

At the beginning of the project, the expectations concerning the benefits of synchronous telemedical consultations were rather high. During the study it became increasingly clear that in those cases where asynchronous telemedicine was used, the factor of satisfaction was rated higher. This was true for both the GPs and the specialist. During the same period, the impact of the COVID-19 pandemic led to a huge increase in the use of video consultation with patients also in Germany. The same applies to the practices that were part of this study [12]. The satisfaction with asynchronous care of dermatology cases is in line with the international findings on which this study was based and has led to a current follow-up project focusing on the asynchronous use of telemedicine [15,16,17]. Another benefit of asynchronous telemedicine is the fact that this form of telemedicine is far easier to implement in everyday practice. The GPs and specialists do not have to attend a video liaison consultation at the same time and most reasons for consultation allowed for a period of 48 h until a response was needed to be provided.

The high level of satisfaction of MFAs during delegated home visits seems to be good evidence of the quality and acceptance of those visits [19,32]. Adding the option of direct telemedical transmission of vital signs to the GP practice to these visits seems unnecessary. Further evidence seems to be the fact that there was no video conferencing with the doctors during the project phase and that an additional visit by the doctor was only necessary in 4 out of 158 cases. In these instances, contact was made via telephone.

### 4.1. Lessons Learned

After the two-year pilot study, we have compiled some recommendations for other studies in the field of implementation and evaluation of TAs in GP practices.

1. Checking the availability of broadband internet access, both mobile and stationary seems trivial today, but makes still sense especially in rural areas, in order to eliminate a frustration barrier.
2. Examining the regional situation helps to avoid investing effort in the implementation of unnecessary TAs. Not every application is suitable for every practice due to regional circumstances [33].
3. Applications should be market-ready, robust, and easy to use. Time for identifying and testing those applications is well spent [5,34].
4. Allow enough time for testing and training the use of the applications. A well-developed training concept right at the beginning of the implementation helps immensely.
5. Think about a technical support plan. If the TA does not work in a used case and no immediate support is available, the willingness to use it will drop sharply.
6. Regular contact with the practices, ideally using the very same technology helps to further deepen the know-how initially gained in the training sessions and generates confidence in the use of the technology [35].
7. Does it work asynchronously? Our pilot study suggests that asynchronous TAs are easier to implement in the workflow of practices. The fact that two doctors do not have to be available online at the same and the lesser demand of broadband internet time makes implementation much easier.

### 4.2. Limitation

In this study, we only evaluated the health care professionals´ views on telemedicine applications, as they were the only ones actively using the applications. No data on the patients’ perspective was collected. Future studies should include the patients’ perspective. 

Since this is a pilot study with twelve practices, one can assume a selection bias among the users. It stands to reason that these practices are early adopters with a high intrinsic motivation for the usage of TAs.

## 5. Conclusions

This pilot study has shown that telemedical care may be implemented into practice when existing, stable technology is used. Especially asynchronous TA could play a relevant role in optimizing access to care in a rural area with limited internet access. Furthermore, with the lessons learned, we are able to present recommendations to assist the implementation of TAs that are transferable to other projects.

## 6. Outlook

As many technical and organizational challenges of telemedicine have now been well studied, the focus of research should shift to the quality of telemedical care, as in our experience research concerning this aspect is still in its infancy [36,37] but is demanded by the political decision-makers at the same time [9].

## Figures and Tables

**Table 1:** Sociodemographic of patients.

TA	Gender	Age (Min–Max)	Age (Average)
Video liaison consultations (N = 17)	♀ 75% ♂ 25%	5–81	51
Teledermatological Consultations (N = 24)	♀ 62% ♂ 38%	5–90	42
Digital vital sensors (N = 158)	♀ 56% ♂ 44%	81–101	87

## Data Availability

Due to ethical consideration no patient data can be shared publicly.
